# Inhibitor of apoptosis proteins, NAIP, cIAP1 and cIAP2 expression during macrophage differentiation and M1/M2 polarization

**DOI:** 10.1371/journal.pone.0193643

**Published:** 2018-03-08

**Authors:** Virginia Morón-Calvente, Salvador Romero-Pinedo, Sofía Toribio-Castelló, Julio Plaza-Díaz, Ana C. Abadía-Molina, Domingo I. Rojas-Barros, Shawn T. Beug, Eric C. LaCasse, Alex MacKenzie, Robert Korneluk, Francisco Abadía-Molina

**Affiliations:** 1 Department of Cell Biology, University of Granada, Granada, Spain; 2 Biomedical Research Centre, University of Granada, Granada, Spain; 3 Department of Biochemistry and Molecular Biology III and Immunology, University of Granada, Granada, Spain; 4 Department of Biochemistry and Molecular Biology II, University of Granada, Granada, Spain; 5 Institute of Nutrition and Food Technology “José Mataix”, University of Granada, Granada, Spain; 6 Institute of Parasitology and Biomedicine “López-Neyra”, Spanish National Research Council (CSIC), Granada, Spain; 7 Apoptosis Research Centre, Children’s Hospital of Eastern Ontario Research Institute, University of Ottawa, Ottawa ON, Canada; 8 Department of Pediatrics, University of Ottawa, Ottawa ON, Canada; 9 Department of Biochemistry, Microbiology and Immunology, University of Ottawa, Ottawa ON, Canada; University of Michigan Health System, UNITED STATES

## Abstract

Monocytes and macrophages constitute the first line of defense of the immune system against external pathogens. Macrophages have a highly plastic phenotype depending on environmental conditions; the extremes of this phenotypic spectrum are a pro-inflammatory defensive role (M1 phenotype) and an anti-inflammatory tissue-repair one (M2 phenotype). The Inhibitor of Apoptosis (IAP) proteins have important roles in the regulation of several cellular processes, including innate and adaptive immunity. In this study we have analyzed the differential expression of the IAPs, NAIP, cIAP1 and cIAP2, during macrophage differentiation and polarization into M1 or M2. In polarized THP-1 cells and primary human macrophages, NAIP is abundantly expressed in M2 macrophages, while cIAP1 and cIAP2 show an inverse pattern of expression in polarized macrophages, with elevated expression levels of cIAP1 in M2 and cIAP2 preferentially expressed in M1. Interestingly, treatment with the IAP antagonist SMC-LCL161, induced the upregulation of NAIP in M2, the downregulation of cIAP1 in M1 and M2 and an induction of cIAP2 in M1 macrophages.

## Introduction

The innate immune system is the first line of defense against external pathogens. The innate immunity response to pathogens is nonspecific and largely depends on macrophages. Macrophages are a heterogeneous cell population that also participates in tissue homeostasis, including the removal of apoptotic cells and cellular debris and in tissue remodelling and repair [1, 2]. Due to their multifunctional roles, macrophages are highly plastic and can modify their metabolism and phenotype in response to microenvironmental cues. Two principal polarization states have been described, M1 macrophages, or classically activated macrophages, and M2 macrophages, or alternatively activated macrophages [3, 4]. M1 macrophages exhibit a pro-inflammatory response, with a high production of effector molecules (reactive oxygen species and nitrogen intermediates) and immunostimulatory cytokines (TNF*α*, IL-1*β* and IL-6). Due to their cytotoxic activity, microbicidal and tumoricidal properties, M1 macrophages are mostly associated with cell-mediated immune responses. On the other hand, M2 macrophages are characterized by a high level of scavenger-, mannose- and galactose-type receptors, and they have an important role during allergies and helminth-driven inflammatory reactions [5, 6]. The dysregulation of macrophage polarization is implicated in the development of pathologies [7], such as diabetes [8, 9], cancer [10–13], atherosclerosis [14, 15], myocardial infarction [16], obesity [17] and asthma [18]. Thus, there is a growing interest in understanding the balancing of M1/M2 polarization and for the possible therapeutic modulation of M1 and M2.

The inhibitor of apoptosis (IAPs) family are required for multiple cellular processes, such as apoptosis, cellular proliferation, cytokinesis, [19, 20], signal transduction, heavy metal homeostasis [21, 22], and immunity [23, 24]. The IAP family members are characterized by the presence of at least one BIR (Baculovirus inhibitor of apoptosis repeat) domain that facilitate protein-protein interactions. As part of the immune response against pathogens, monocytes that are differentiating into macrophages undergo apoptotic stress [25]. In addition, the cellular IAP 1 (cIAP1) is involved in the secretion of proinflammatory cytokines in macrophages and is redistributed from the nucleus to the cytoplasm during PMA-induced differentiation of monocytes to macrophages [26, 27]. Furthermore, NAIP participates in the formation of the NLRC4 inflammasome, a signaling platform that, upon binding of a pathogen-associated molecular pattern (PAMP) ligand to NAIP, recruits and activates caspase-1, a proteolytic enzyme that processes the precursors of interleukin-1*β* and interleukin-18 cytokines for extracellular secretion. [28–30].

The expression profiles of the IAPs, namely cIAP1, cIAP2 and NAIP during the differentiation of monocytes to macrophages and in polarization into M1/M2 states is unknown. Moreover, the functional roles of the IAPs in modulating these processes is also unknown. The aim of this study is to examine the differential expression of the most immunologically relevant IAPs during monocyte-to-macrophage differentiation and polarization; an analysis that will help in setting direction for future studies aimed at the functional and molecular dissection of the IAPs roles in these critical transitions. We chose to work with two different lineage models, the monocytic human cell line THP-1, which can be *in vitro* differentiated into macrophages [31], and also with human peripheral blood monocytes from healthy donors.

## Materials and methods

### Cell culture, differentiation and polarization

The use of human samples was approved by the *“Comité de Ética en Investigación Humana”* of the Granada University. Approval number 417. Informed consent was obtained from all the participants.

Human myeloid leukemia THP-1 cells (obtained from the *Centro de Instrumentación Científica*, University of Granada, Spain) were grown in RPMI 1640 (Lonza, Allendale, NJ) supplemented with 10% of heat inactivated fetal calf serum (GIBKO, California, USA), 1mM of L-glutamine (PAA) and 1% of penicillin-streptomycin (Cambrex, Bio Science) in standard conditions (37°C in 5% CO2 humidified atmosphere). THP-1 monocytes were differentiated into M0 macrophages by 24h incubation with 10ng/mL of phorbol 12-myristate 13-acetate (PMA, Sigma-Aldrich) followed by 24h of culture in standard RPMI 1640 media. THP-1 M0 cells were polarized towards M1 macrophages by treatment with 100ng/mL of Lipopolysaccharide (LPS) and 20ng/mL of Interferon-*γ* (IFN-*γ*) for 48h. THP-1 M2 macrophages were generated by 48h treatment with 20ng/mL of IL-4.

Isolation of human primary blood monocytes. Peripheral blood mononuclear cells (PBMCs) were isolated from buffy coats of four healthy donors by density-gradient centrifugation using Ficoll-Histopaque (Sigma-Aldrich, St Louis, MO), followed by immunomagnetic separation according to manufacturer’s instructions (Dynabeads Untouched Human Monocytes Kit from Invitrogen). The purity of the separation was confirmed measuring CD14 positive cells by flow cytometry (>to 95%). Differentiation of monocytes into resting macrophages occurred 7 days after culture in RPMI 1640 supplemented with 10% of heat inactivated fetal calf serum, 1mM of L-glutamine and 1% of penicillin-streptomycin in standard conditions (37°C in 5% CO2 humidified atmosphere). Polarization into M1 was accomplished by maintaining the standard monocyte culture for 5 days (change of media on day 4), then treated with 20ng/mL of INF-*γ* and 1 hour later with 100ng/mL of LPS for 48h. M2 polarization was induced by culturing monocytes for 6 days in standard conditions and then maintained 24h in the presence of 20ng/mL of IL-4. Written informed consent was obtained from all participants (University of Granada *Comité de Ética en Investigación Humana*; #417).

### Gene expression analysis

Total RNA was extracted from cells with Trizol (Invitrogen) as recommended by the supplier. cDNA was obtained using the Promega Reverse Transcription System kit according to manufacturer’s instructions. The synthesized cDNA was, then used for semi-quantitative PCR or real-time quantitative PCR. For the semi-quantitative PCR, PCR mastermix of Promega was employed and PCR amplification was performed in a 2720 Thermal Cycler Geneamp (Applied Biosystems) under the following conditions: after a first step of inactivation at 95°C for 5 min, 30 cycles of 94°C for 45 sec, 50°C for 1 min, 72°C for 45sec and a final extension step at 72°C for 5 min. All PCR products were analyzed by electrophoresis on 1% agarose gel, calibrated with, photographed and quantified by densitometric scanning using *ImageJ* program (National Institutes of Health, USA). The real-time quantitative PCR was performed employing the SsoAdvanced SYBR Green supermix (Biorad) on a Mastercycler RealPlex2 (Eppendorf) using the Realplex software. PCRs were conducted using the primers shown in Table 1.

**Table 1:** Pairs of primers used for mRNA determinations.

Heading1		
Primers	Forward (5’→3’)	Reverse (5’→3’)
hNAIP exon 4	GCTCATGGATACCACAGGAGA	CTCTCAGCCTGCTCTTCAGAT
hNAIP exon 16-17	GAATTTATCGAGTGGCCAAAC	TCAAAGACTTGACTGTTGTGG
hCXCL10	GAAAGCAGTTAGCAAGGAAAGGTC	ATGTAGGGAAGTGATGGGAGAGG
hCD14	ACAGGTGCCTAAAGGACTGC	GATTCCCGTCCAGTGTCAGG
hCD18	CAGCTCACTCTGACCACTTCT	TCTGCCAGGAGGTATAGACGA
hCD163	GTCGCTCATCCCGTCAGTCATC	GCCGCTGTCTCTGTCTTCGC
hCD206	ACCTCACAAGTATCCACACCATC	CTTTCATCACCACACAATCCTC
hActin	TGACGGGGTCACCCACACTGTGCCCATCTA	CTAGAAGCATTTGCGGTGGACGATGGAGGG
hHPRT1	TGACACTGGCAAAACAATGCA	GGTCCTTTTCACCAGCAAGCT
hGAPDH	TGCACCACCAACTGCTTAGC	GGCATGGACTGTGGTCATGAG
human cIAP1, cIAP2 and XIAP primers were obtained from realtimeprimers.com		

### Western blot analysis

Cells were washed 2 times with cold PBS, scraped and lysed in radioimmunoprecipitation assay (RIPA) lysis buffer containing a protease inhibitor cocktail (Roche) for 30 minutes at 4°C, followed by centrifugation at 13,000g for 15 minutes. Supernatants were collected and kept at -20°C. Total amount of protein was determined using a Bio-Rad protein assay kit. Equal amounts of soluble protein was separated on polyacrylamide gels (7-10%) followed by transfer to nitrocellulose membranes.

Individual proteins were detected by Western blotting using the following antibodies: NAIP-J2 [32] and RIAP1 [33] rabbit polyclonal antibodies were used to detect human NAIP and cIAP1/2 respectively. HSC-70 (sc-7298) from Santa Cruz biotechnology were used as loading control. AlexaFluor680 (Invitrogen) or IRDye800 (Li-Cor) were used to detect the primary antibodies, and infrared fluorescent signals were detected using the Odyssey Infrared Imaging System (Li-Cor). Quantification was performed by densitometry analysis using the *ImageJ* program (National Institutes of Health, USA).

### Flow cytometry

Cells were washed 2 times with cold PBS, scraped and collected by centrifugation and resuspended in FACS buffer (PBS supplemented with 0,5% bovine serum albumin and 5mM of EDTA). Cells were stained with anti-CD11b (clone ICRF44, FITC conjugated; Biolegend) or isotype control (mouse IgG1 FITC conjugated biolegend);, anti-CD14 (clone M5e2, APC conjugated; Biolegend) or isotype control (mouse, IgG2a, *κ*, APC conjugated, Biolegend), anti-CD206 (clone 15-2, APC/cy7 conjugated; Biolegend) or isotype control (mouse IgG1, APC/cy7 conjugated, Biolegend), anti-CD86 (clone IT2.2, Alexa Fluor 488 conjugated; Biolegend) or isotype control (mouse IgG2b, *κ*, Alexa Fluor 488 conjugated, Biolegend) and anti-CD163 (clone RM3/1, Alexa Fluor 647 conjugated; Biolegend) or isotype control (mouse IgG1, *κ*, Alexa Fluor 647 conjugated, Biolegend) Cells were analyzed on a BD LSRFortessa X-20 Cell Analyzer (BD Bioscience) with post-processing in FlowJo software (Tree star Inc). Cell populations were gated on forward and side scatter to select intact single cells. The gating strategy and a representative flow diagram is shown in S2 Fig.

### Immunostaining and microscopy

Macrophages were grown in 2-well glass chambered coverslip (ibidi Ca#80286). Cells were fixed for 10 minutes in ice-cold 2% paraformaldehyde in PBS, briefly rinsed in PBS and permeabilized with 0.2% Triton X-100/PBS for 10 minutes. Cells were incubated overnight at 4°C with the primary antibody diluted in PBS (abcam ab98020, human NAIP, 1:200), rinsed 3 times for 5 minutes with PBS and incubated for 50 minutes at room temperature with the secondary antibody (goat anti-mouse Alexa Fluor 488, Invitrogen, A-11070, diluted at 1:1000 in PBS). The slides were counterstained for 5 minutes with Hoechst 33342 (Invitrogen) diluted at 10*μ*g/ml in PBS. The coverslips were then rinsed 3 times for 5 minutes with PBS and mounted with ProLong Gold (Invitrogen). Confocal microscopy was performed with a Nikon Eclipse Ti-E microscope. Mean fluorescence intensity per pixel per cell in resting and polarized macrophages was determined using using the *ImageJ* program (National Institutes of Health, USA).

### Statistical analysis

Statistical analysis was performed with Graphpad Prism 5 software. Unpaired 2-tailed Student’s t-test was used to compare data sets consisting of 2 treatment groups. One-way ANOVA with a Bonferroni *post hoc* test was used to calculate the levels of significance between multiple groups. All THP-1 results were obtained with a minimum of three independent experimental replications and PBMC results were obtained from samples from four healthy individuals.

## Results

### Establishment of the THP-1 macrophage polarization model

**Fig 1:**
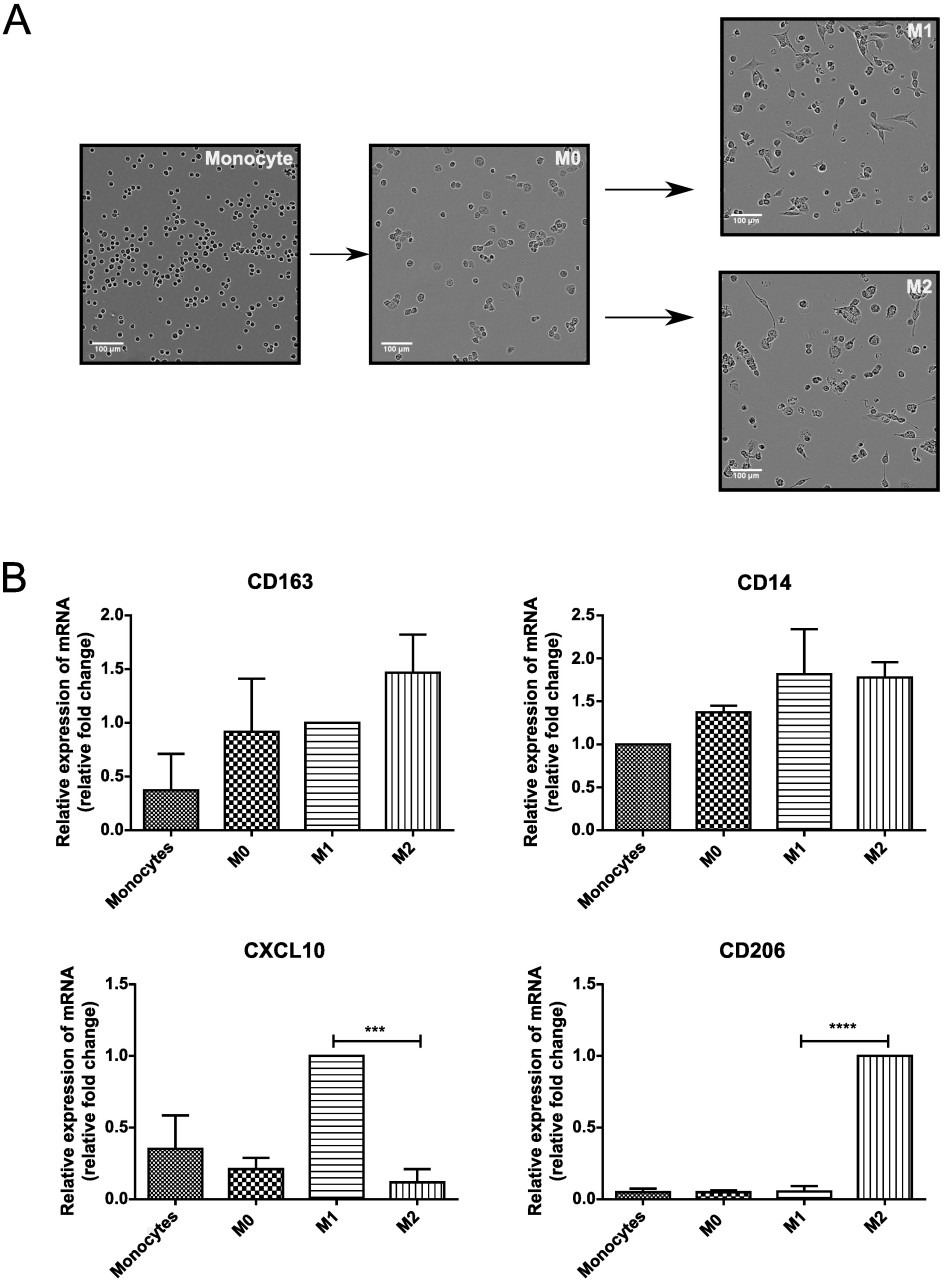
Validation of the macrophage differentiation and polarization THP-1 model. THP-1 monocytes were incubated with 10ng/mL PMA for 24 h to obtain M0 macrophages and then cultured in regular media for 24 h. Cells were then treated with 20 ng/mL IFN*γ* and 100 ng/mL LPS or 20 ng/mL IL-4 for 48 h for the induction of the M1 or M2 states, respectively. A: Phase contrast microscopy images of THP-1 monocytes, and M0-, M1- and M2-macrophages. B: The expression of the indicated genes was examined by RT-qPCR. Values are normalized to internal controls (HPRT1 and GAPDH) in each group and then the fold change was calculated taking the expression in M1 as the base line in the study of CXCL10 and CD163, M2 expression as the base line in the CD206 analysis and monocyte expression as the baseline in CD14 analysis. Data represent the mean and standard deviation of three independent experiments. *** P<0.001 (ANOVA with Bonferroni post hoc).

The THP-1 cell line has been amply used for *in vitro* studies of monocyte-to-macrophage differentiation [34, 35] and as a model for macrophage polarization [36–38]. We adapted and optimized these protocols for macrophage differentiation and polarization into M1/M2 subsets. We obtained THP-1 macrophages by initially treating cells with 10ng/mL of PMA (24 hours) and then cultivating with fresh media, without PMA for 24 hours to allow full differentiation into macrophages (denoted M0). Polarized cells were obtained either by culturing M0 cells for 48 hours with 100ng/mL LPS and 20ng/mL INF*γ* to obtain classical activated macrophages (M1) or with 20ng/mL IL-4 to acquire an alternative activation phenotype (M2). Under these conditions, THP-1 cells demonstrated typical macrophage morphological changes (Fig 1A). Undifferentiated THP-1 cells are non-adherent and have a round shape, while PMA treated cells become adherent with a flat and amoeboid shape. M1 and M2 polarized THP-1 cells presented typical cellular protrusions of an activation state, including lamellipodia and filopodia [39]. To confirm macrophage differentiation and polarization, we measured gene expression of the macrophage markers CD14 and CD163 and of the M1/M2 markers, respectively, CXCL10 and CD206. We observed a trend, albeit not statistically significant, of the expression of CD163 and CD14 upon differentiation of THP-1 cells into M0 (Fig 1B). However, we observed a significant increase in the expression of CXCL10 in THP M0 cells treated with LPS and INF-*γ* and a significant increase of CD206 expression in M0 cells treated with IL-4. Together, these results confirm that we are able to differentiate THP1 cells into macrophages and to further induce polarization of these cells into M1 or M2 states.

### Expression of the IAPs in monocytes and polarized macrophages

#### NAIP expression in M0, M1 and M2 THP-1 cells

**Fig 2:**
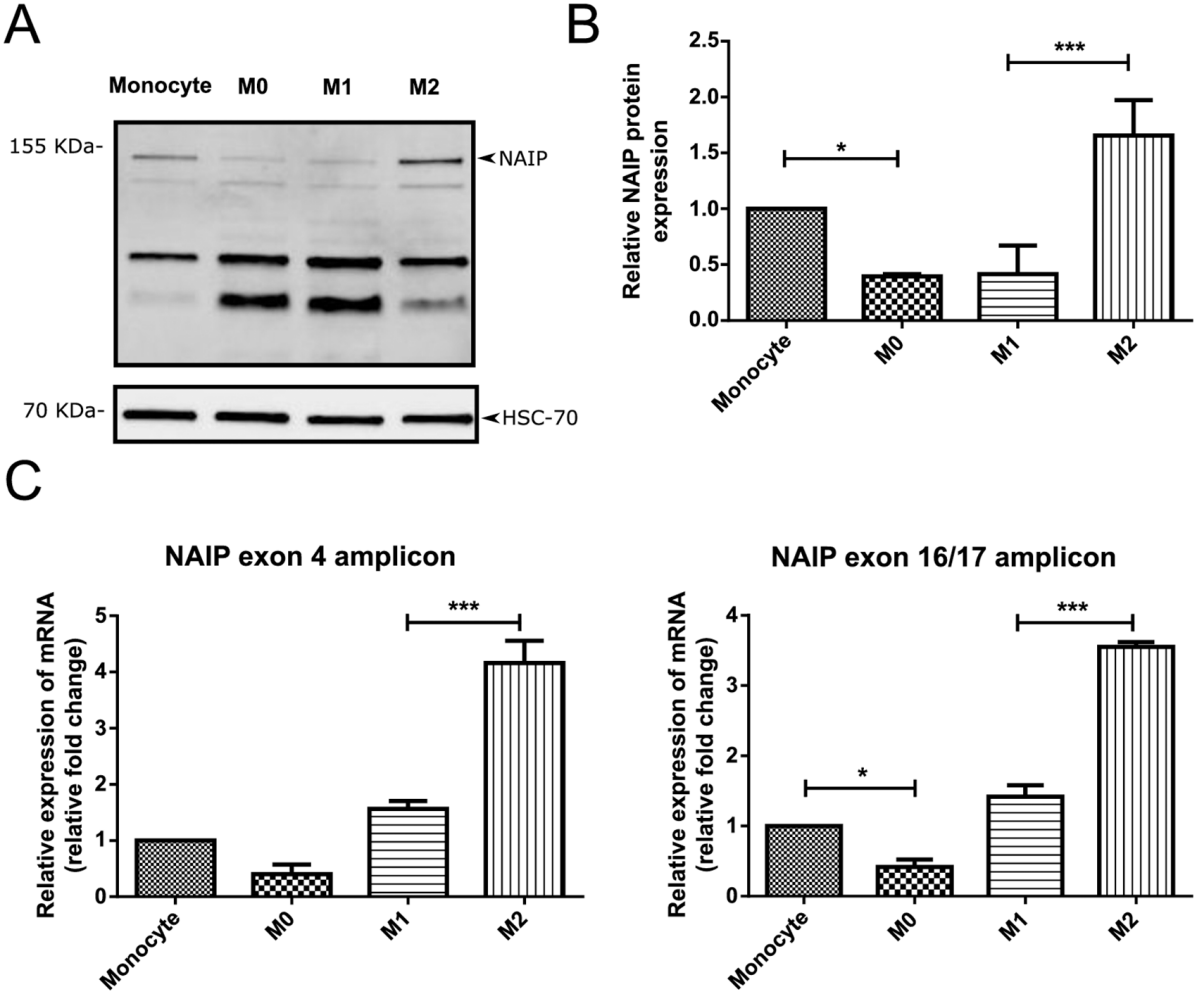
NAIP expression in monocytes, M0-, M1- and M2-macrophages. A, B: The protein level of NAIP was assessed by western blotting of cell extracts from undifferentiated monocytes and M0 or polarized M1/M2 THP-1 macrophages. HSC-70 was used as loading control. A: Representative western blot of one of three experiments with similar results. B: Quantification of NAIP protein abundance in western blots normalized to HSC-70 of three independent experiments. C: NAIP mRNA expression of NAIP was was analysed by RT-qPCR using primers that span exon 4 or between exons 16 and 17 of NAIP. Values are normalized to internal controls (HPRT1 and GAPDH) and presented as mean values ± s.d. of fold-change of three independent experiments. *P<0.05 ***P< 0.001 (ANOVA with Bonferroni post hoc).

The expression profile of NAIP in monocytes and macrophages is currently unknown. Western blot and RT-qPCR analysis were performed to characterize the expression of NAIP during the differentiation of THP-1 monocytes into M0 and M1/M2 polarized macrophages. We observed that the protein levels of NAIP were significantly downregulated upon differentiation into M0 macrophages and that the expression of NAIP was significantly upregulated in the M2 subset (Fig 2A and 2B). We observed a similar trend of NAIP mRNA expression, using primers that amplify regions at exon 4 and a region between exons 16 and 17 (Fig 2C).

### Analysis of NAIP expression in PBMC-derived M1/M2 macrophages

**Fig 3:**
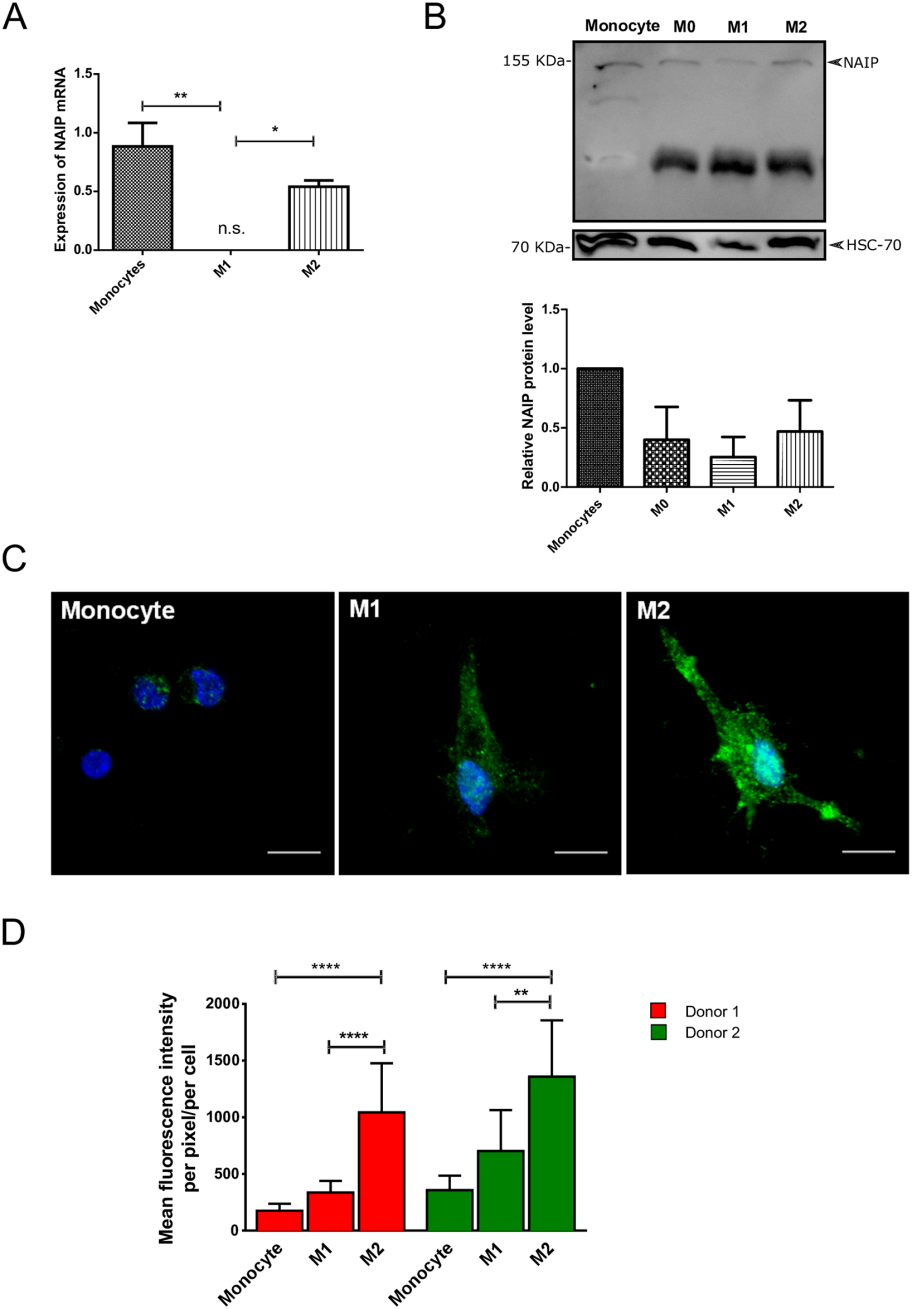
NAIP expression in polarized primary human macrophages. A: NAIP mRNA levels from PBMC-derived monocytes or M1 and M2 macrophages were analyzed by RT-qPCR. Values are normalized to internal controls (HPRT1 and GAPDH) and presented as mean values ± s.d. of fold-change of three independent experiments. B: Protein levels of NAIP in the indicated cell populations were assessed by western blotting. Representative western blot from samples of one of the donors. Graph represents the quantification of the NAIP protein, normalized to HSC-70 of experiments from four different healthy donors. C: Representative confocal microscopy images of monocytes, M1 and M2 macrophages from volunteer 1. Note the amoeboid-like shape in M1 and M2 macrophages. Bar, 10*μ*m. D: Mean fluorescence intensity in monocytes and M1 and M2 macrophages from two healthy donors. 10 randomly selected cells in each case were analyzed for their mean fluorescence intensity per cell area using the *ImageJ* program. *Significant difference (P = 0.0163), **significant difference (P = 0.0037), ***significant difference (P = 0.0005).

We next assessed whether the expression pattern of NAIP in polarized THP-1 macrophages is consistent in primary human macrophages. Prior to the analysis of NAIP expression, we confirmed that PBMC monocytes can be polarized to M1 or M2 macrophages. As expected, the expression of M2 markers, CD206, CCL18 and CD163, considered both an M0 and an M2 marker, were significantly upregulated after IL-4 treatment and the expression of the M1 marker CXCL10 was only present in macrophages treated with LPS and INF-*γ* (S1 Fig). For the analysis of NAIP expression, we observed that the mRNA level of NAIP was similar in monocytes and M2 macrophages, but was significantly downregulated in M1 macrophages (Fig 3A). However, while we did not observe an statistical difference in the protein levels of NAIP between monocytes and M0 or polarized macrophages (P = 0.363, Fig 3B), we observed a significant increase of NAIP levels in M2 macrophages by immunofluorescence (Fig 3C and 3D); with respect to NAIP expression in polarized macrophages we want to emphasized that, lower NAIP expression in M1 cells with greater NAIP levels in the M2 state are shown both in THP-1 and PBMCs derived M1/M2 macrophages with a good correlation in NAIP mRNA and protein levels by western blot (Figs 2 and 3A and 3B).

#### cIAP1 and cIAP2 expression in M0, M1 and M2 cells

**Fig 4:**
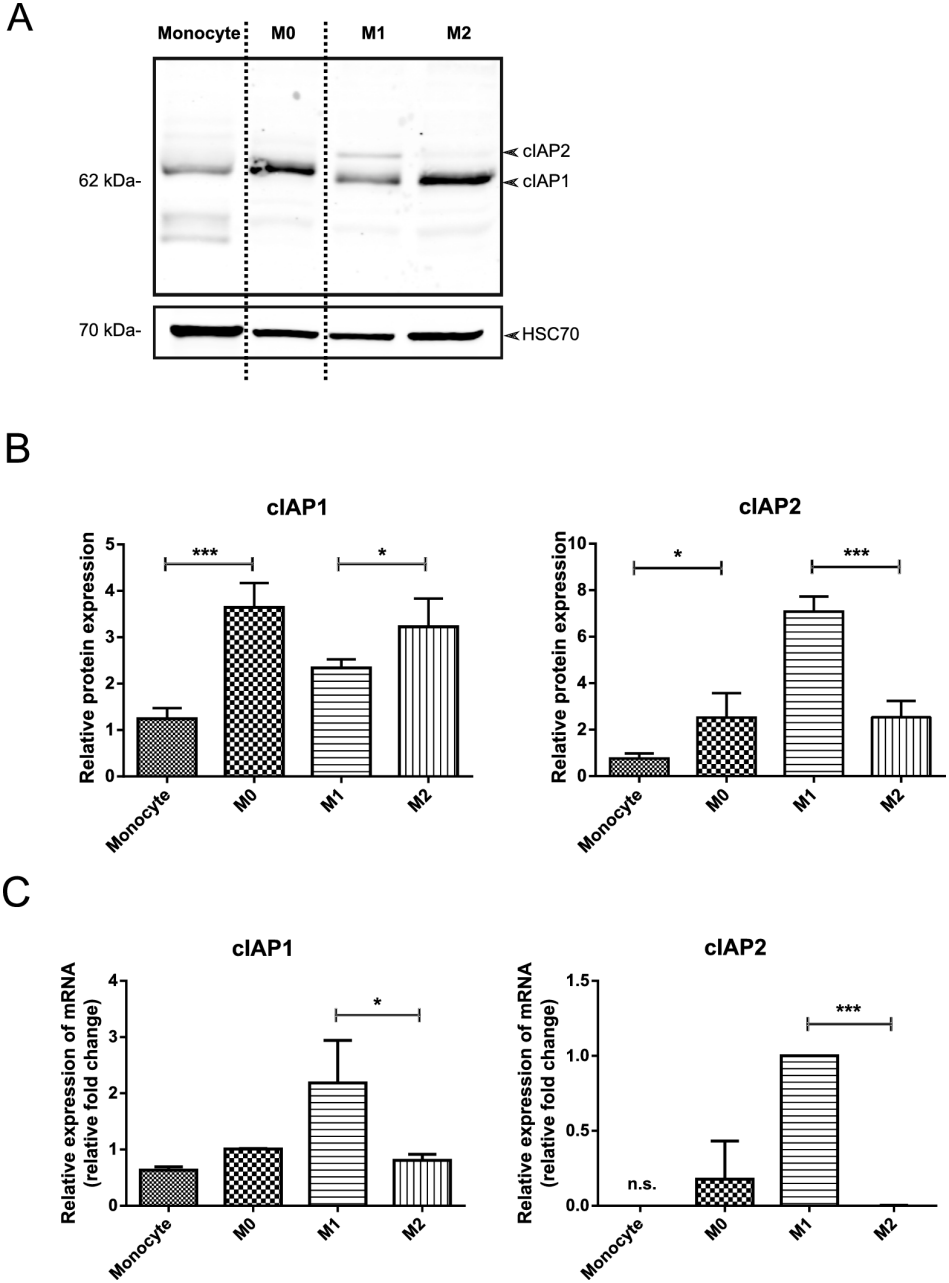
cIAP1 and cIAP2 expression in unpolarized (THP-1/monocytes and M0-) and polarized M1- and M2-macrophages. A, B: Polarized and unpolarized THP-1 cell proteins were extracted and cIAP1/2 protein abundance was assessed by western blotting using the RIAP1 antibody. HSC-70 was used as loading control. A: Representative western blot of one of three experiments with similar results. B: Quantification of cIAP1 and cIAP2 protein abundance in western blots normalized to HSC-70 of three independent experiments. C: Total RNA was extracted, reverse transcribed and cIAP1 and cIAP2 mRNA were analysed by RTq-PCR. Values are normalized to internal controls (HPRT1 and GAPDH) and presented as mean values ± s.d. of fold-change of three independent experiments. *P<0.05 ***P< 0.001 (ANOVA with Bonferroni post hoc).

We next determined the expression profiles of the cellular IAPs, cIAP1 and cIAP2 in THP-1 monocytes, M0- and polarized-macrophages. We found that the cIAP1 protein expression level was significantly increased following PMA-induced differentiation of monocytes into M0 and that that level of expression was maintained in M2 macrophages (Fig 4A and 4B). However, the mRNA levels of cIAP1 in polarized macrophages did not correlate with the protein levels of cIAP1 (Fig 4C), indicating that there may be post-transcriptional or post-translational processes that regulate cIAP1 protein levels. On the other hand, we observed consistent upregulation of cIAP2 mRNA and protein levels in M1 macrophages (Fig 4A, 4B and 4C).

### Effect of IAP inhibitors during macrophage polarization

**Fig 5:**
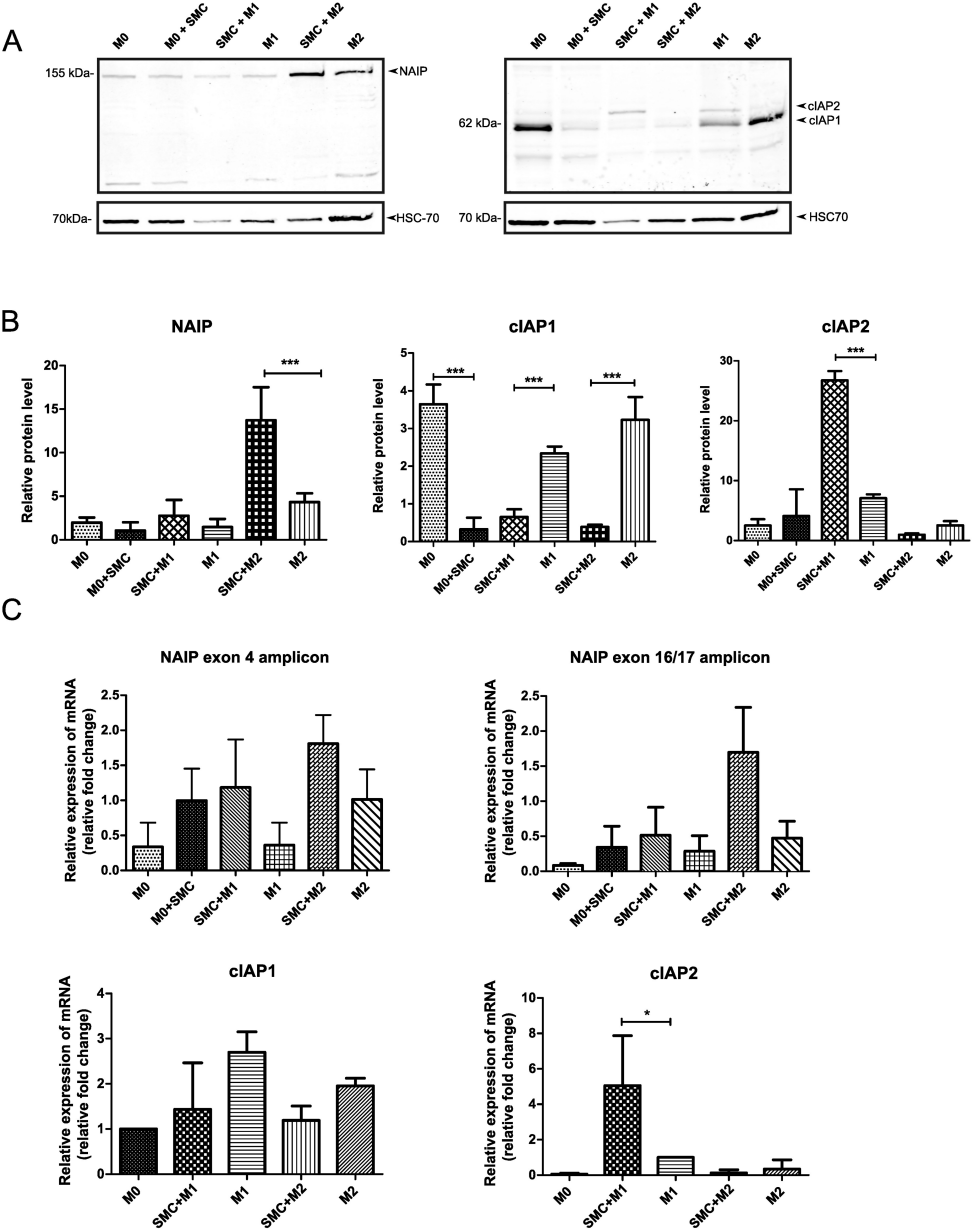
Smac mimetics modulate IAP expression during macrophage polarization. THP-1 M0 macrophages were treated with 1*μ*M LCL161 for 24 h prior to polarization into M1 or M2. A, B: The levels of NAIP, cIAP1 and cIAP2 were determined by western blotting. HSC-70 was used as loading control. A: Representative western blot from one of three experiments with similar results. B: Quantification of NAIP, cIAP1 and cIAP2 protein abundance normalized to the corresponding HSC-70 in three independent experiments. C: Determination of NAIP, cIAP1 and cIAP2 mRNA levels by RT-qPCR. NAIP mRNA expression was evaluated using primers spanning exon 4 or between exons 16 and 17. Values are normalized to internal controls (HPRT1 and GAPDH) and presented as mean values ± s.d. of fold-change of three independent experiments. *P<0.05 **P<0.01 ***P< 0.001 (ANOVA with Bonferroni post hoc).

SMAC mimetic compounds (SMCs) are a class of small molecules that mimic the structure of the endogenous Second mitochondrial activator of caspases (SMAC). SMCs are IAP antagonists, whereby they inhibit the function of cIAP1, cIAP2 and XIAP [40]. We next determined whether IAP antagonism through the use of SMC-LCL161 can modulate polarization of macrophages into M1 or M2 [41, 42]. We examined whether SMC treatment affected the expression levels of NAIP, cIAP1 and cIAP2 in M0 macrophages exposed to LCL161 prior to stimulation into M1 or M2 states. We found that pre-polarization treatment with LCL161 significantly enhances NAIP levels in M2 macrophages (Fig 5A and 5B). We observed a similar trend, albeit not statistically significant, of the mRNA levels of NAIP by RT-qPCR using primers that span exons 16 and 17 or exon 4 (Fig 5C). A property of SMCs, including LCL161, is the downregulation of cIAP1 and cIAP2 [43]. We consequently analyzed the levels of cIAP1 and cIAP2 in treated M0 and polarized macrophages pre-treated with LCL161. We found a reduction in the protein levels of cIAP1 in LCL161-treated M0, M1 or M2 cells (Fig 5A and 5B). On the other hand, we observed elevated protein levels of cIAP2 in LCL161-treated M1 cells (Fig 5A and 5B). Similarly, cIAP1 and cIAP2 mRNA expression in SMC pre-treated M1 and M2 cells matched the corresponding protein levels (Fig 5C).

**Fig 6:**
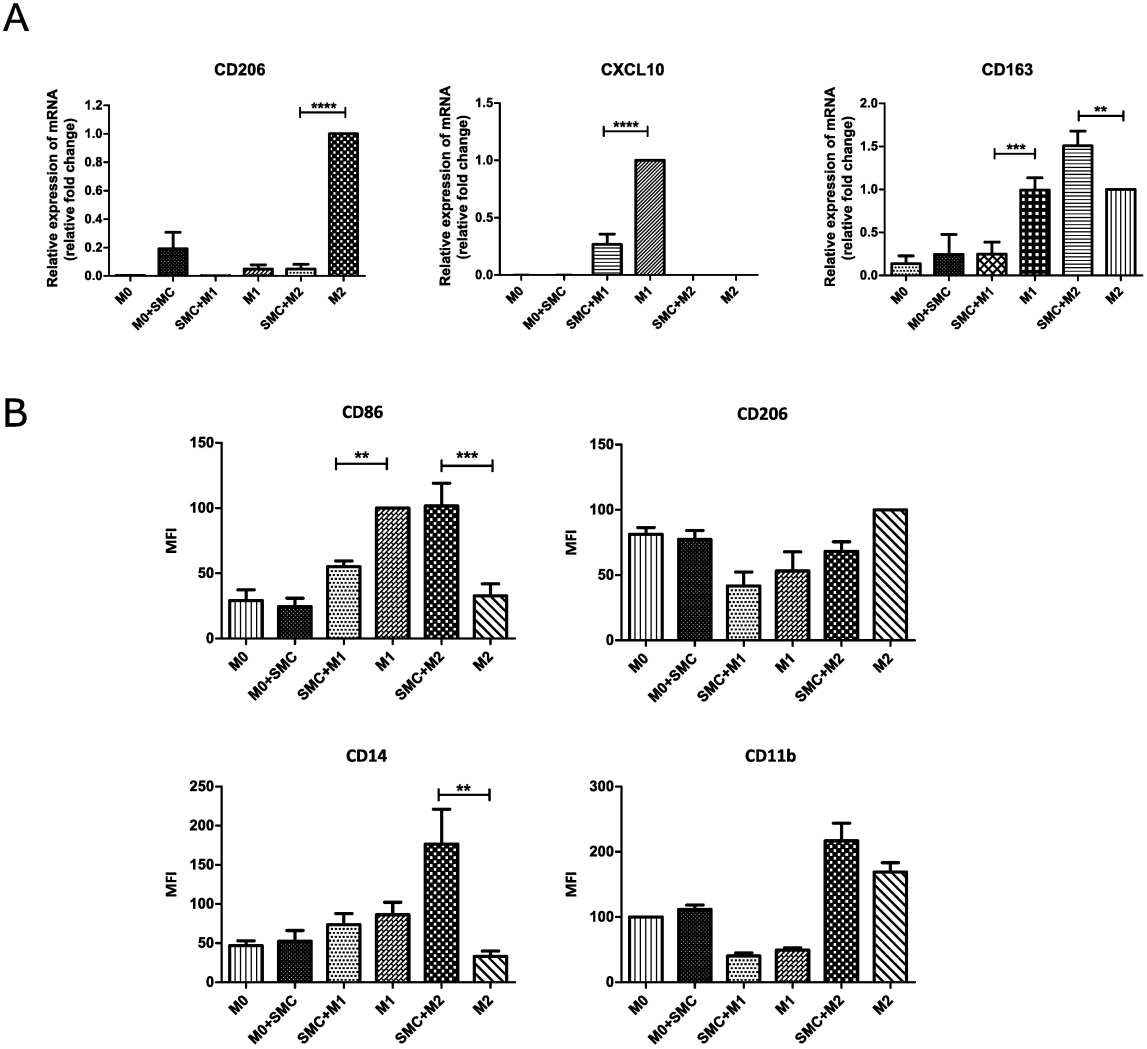
SMCs alters the markers expression profile of polarized macrophages. THP-1 M0 macrophages were treated with 1*μ*M LCL161 for 24 hours and then induced to polarized into M1 or M2 states. A: CD206, CXCL10 and CD163 polarization marker genes were examined by semi-quantitative RT-PCR. mRNA expression was normalized to GAPDH in each group and then calculated as fold change against the expression of the control group. Data represent the mean and standard deviation of three independent experiments. B: Cells were subsequently analyzed by flow cytometry for the expression of the macrophage markers CD14 and CD11b and of the M1 (CD86) and M2 (CD206) polarization markers. Data represents mean MFI of three independent experiments. *P<0.05 *** P<0.0001 (ANOVA with Bonferroni post Hoc).

Given the differences in NAIP and cIAP2 expression in SMC-treated polarized macrophages, we next assessed whether SMC treatment affects macrophage polarization into M1 or M2 by flow cytometry and RT-qPCR. We observed an attenuated expression of the M1 marker CD86 in THP-1 macrophages stimulated into the M1 state which is concomitantly upregulated under conditions that induce polarization into M2 (Fig 6B). Consistent with these results, we observed a downregulation of CXCL10, a chemokine commonly associated with M1 [36], in SMC treated THP1 M1 cells (Fig 6A). On the other hand, we observed complete ablation of the M2 marker CD206 in SMC treated M2 cells (Fig 6A). Interestingly, when we profiled the expression of macrophage markers, such as CD14 and CD163, we observed consistent upregulation of these markers in LCL161-treated M2 THP-1 cells and a similar trend for CD11b (Fig 6A and 6B), which is consistent with the enhanced levels of NAIP (Fig 5) in SMC treated THP-1 M2 cells. The significance of this upregulation of NAIP and macrophage markers upon antagonism of the IAPs will need to be analyzed in future studies.

## Discussion

The IAPs have been characterized to be involved in several immunological processes. However, the differential expression of the IAPs in polarized macrophages remained unexplored. Here we show the expression pattern of NAIP, cIAP1 and cIAP2 during differentiation into macrophages and polarization into M1/M2 and after the use of the IAP antagonist, monovalent SMAC mimetic compound LCL161, in macrophages prior to polarization stimulation.

Macrophage polarization is critical for tissue homeostasis. In certain circumstances, macrophages can adopt into a proinflammatory state, acting mainly as a defense against external pathogens or participating in the repair of damaged tissue. Particular M1/M2 profiles have been associated with pathologies in which a polarization state switch is associated with the disease [4, 7]. In cancer, tumor-associated macrophages usually present an M2 phenotype and are believed to help tumor progression and dissemination of the cancer cells [44, 45]. M2 macrophages have been implicated in the pathogenesis of tuberculosis [46, 47] and in the induction of type 2 diabetes caused by obesity [48]. A predominant M1 polarization state has been connected to bipolar disorder [49] and the development of nonalcoholic fatty liver disease [50, 51]. Furthermore, the presence of M1 macrophages within the tumor microenvironment is associated with the elimination of tumors following combinatorial anticancer therapy strategies [12, 13]. Clearly, the investigation and further understanding of the mechanisms leading to macrophage polarization and the switch between M1/M2 states are of current interest and might contribute to novel therapeutic approaches.

In this study, we observed distinctive IAP expression profile patterns characteristic to the M1 or M2 macrophage polarization states. We observed that NAIP expression is subdued in classically activated macrophages (a.k.a., M1), a finding that was unexpected as NAIP functions as a cytosolic biosensor for some PAMPs and is required for formation of the NLRC4 inflammasome complex [52]. Conversely, NAIP expression is most abundant in M2 macrophages. We have recently demonstrated that NAIP is positively involved in the cell division process [20], which is in line with other studies suggesting that M2-like tissue resident macrophages have a self-renewal capacity [53, 54]. Hence, the enhanced expression of NAIP in M2 macrophages proliferation might account for the potential of macrophage proliferation. Interestingly, NAIP expression is increased during adipogenesis [55] and the M2 state has been described as the main macrophage phenotype found in adipose tissue also presenting local proliferation ability [8, 48].

In the analysis of the expression profiles of cIAP1 and cIAP2 in polarized macrophages, we observed upregulation of cIAP2 in M1 macrophages. This finding is consistent with the premise that IFN-*γ* [56] or LPS treatment induce the upregulation of cIAP2 [24, 57], in addition, LPS treatment activates the alternative NF-KappaB pathway, previously reported to upregulate cIAP2 [58].

This finding is also similar to another study in which cIAP2 transcriptional levels are upregulated in M1 macrophages [59, 60]. In our attempts to analyze the expression of cIAP1, we found that cIAP1 gene transcript levels do not correlate with the corresponding protein levels. The levels of cIAP1 mRNA were significantly higher in M1 macrophages, but the protein levels showed higher in M2- and M0-macrophages. A potential reason for this discrepancy is that the induction of M1 state by LPS/IFN-*γ* treatment promotes cIAP1 degradation through MyD88 [61], which is a compensatory gene expression mechanism. To our knowledge, there have not been any studies that report the induction of cIAP1 during treatment of LPS or IFN-*γ*. A further consequence of cIAP1 degradation in M1 macrophages is the resulting increase of cIAP2 stability, as the degradation of cIAP1 promotes non-canonical activation of NF-*κ*B that leads to upregulation of cIAP2 genes [62].

SMAC mimetic compounds constitute a group of small molecules that target cIAP1 and cIAP2, which promote self-ubiquitination and subsequent proteasomal mediated degradation [62]. SMCs are currently in clinical trials for the treatment of cancer [63–65]. Here, we studied the effect of a monovalent SMC, LCL161, during *in vitro* macrophage polarization. The treatment of M0 macrophages with LCL161 leads to the downregulation of cIAP1 in M1 and M2 and induction of cIAP2 in M1 macrophages. The upregulation of cIAP2 may be a consequence of the engagement of the alternative NF-kappaB pathway by the loss of the cIAPs or by the LPS-induced engagement of the alternative NF-kappaB pathway, which induces the expression of cIAP2 [58, 66–68]. As different macrophage polarization states are reported to play different roles in tumorigenesis [11, 69], it is possible that the ability of SMCs to modulate the polarity of macrophages within tumors can be exploited to eradicate tumors. Indeed, IAP antagonism has been shown to promote the presence of M1 macrophages and is postulated to be important for the eradication of tumors in mouse models when used in combination with immunomodulatory anti-cancer therapeutics [13, 70, 71]. Overall, our results imply that either cIAP1 or cIAP2 could have a direct role in the modulation of the polarization state during tissue homeostasis.

We observed that SMC treatment induces the upregulation of NAIP in M2 macrophages. We are intrigued about the elevated level of NAIP in M2 macrophages and that this expression pattern is further enhanced by IAP antagonism. We hypothesize that SMCs do not interact with NAIP as has been reported that SMAC does not antagonize the ability of NAIP to inhibit caspase-9 [72, 73]. It is possible that the induction of NAIP expression is related to engagement of the NF-kappaB pathway; NAIP expression has been shown to be downregulated in cells treated with NF-*κ*B inhibitors [74]. Hence, it is probable that SMC treatment induces activation of the alternative NF-kappaB pathway, which leads to increased NAIP levels in M2 macrophages.

Although *in vitro* studies do not always mimic the *in vivo* situation, we would like to indicate that this is the main reason for us to have explored IAP expression in a human monocytic cell line as well as in primary derived monocytes from healthy donors; we think that the very different lineage in both models somehow strengthens our observations.

## Conclusion

Here we document a previously unknown expression profile in differentiating macrophages and the M1/M2 macrophage polarization states for the most immunologically-relevant IAP proteins, NAIP, cIAP1 and cIAP2. Remarkably, NAIP expression is most abundant in M2 macrophages, while cIAP1 and cIAP2 show an inverse pattern of expression in polarized cells, cIAP2 is preferentially expressed in M1-macrophages and cIAP1 in M2-macrophages. IAP antagonist treatment of resting M0 macrophages preceding polarization stimulation, induced the upregulation of NAIP in M2 and the downregulation of cIAP1 in M1 and M2 but an induction of cIAP2 in M1 macrophages. Future studies will elucidate the mechanistic roles of these IAPs underlying macrophage differentiation and polarization and will lead to a better understanding of the involvement of the IAPs within the M1/M2 phenotype switch, an area that is receiving increasing attention due to the implications of M1/M2 status with diverse pathologies and therapy strategies.

## Supporting information

S1 Fig Expression of polarization markers in primary human monocytes, M0-, M1- and M2-macrophages. Peripheral blood monocytes, M0 macrophages, and M1 and M2 polarized macrophages were harvested and RNA extracted. mRNA expression of the M2 markers CD206 and CCL18, CD206, considered both an M2 and a macrophage marker, and of the M1 marker CXCL10 were analyzed by RT-qPCR. The expression of each marker was normalized to GAPDH in each group and then calculated as fold change against the expression of monocytes. Data represent the mean and standard deviation of three independent experiments. *P<0,05 **P<0,01 *** P<0.001 (ANOVA with Bonferroni post hoc in A and B and Student’s t-test in D were performed).
(PDF)

S2 Fig Flow cytometry gating strategy for THP-1 cells. Cells were first gated for monocytes or macrophages (SSC-A vs FSC-A) and then for singlets (FSC-A vs FSC-H). The expression of different markers (CD11b, CD14, CD163, CD86 or CD206) were then analyzed in the singlets gate by measuring their median fluorescence intensity. The gating strategy for CD86, representative of all the markers analyzed, is shown.
(PDF)

S3 Fig Western blots zip file. Uncropped Western blot images corresponding to figures in the main manuscript.
(ZIP)
