# Supplementary figures and images for: Inhibitor of apoptosis proteins, NAIP, cIAP1 and cIAP2 expression during macrophage differentiation and M1/M2 polarization
Source: PLoS One. 2018 Mar 8;13(3):e0193643. doi: 10.1371/journal.pone.0193643 (PMC5843221; doi:10.1371/journal.pone.0193643)

**A****CD206**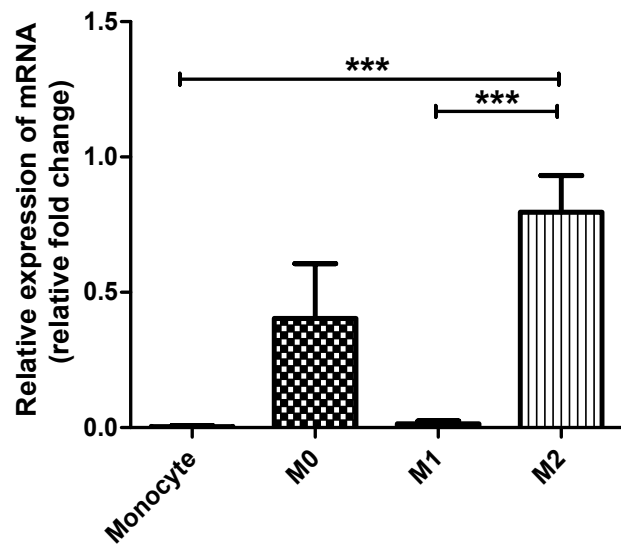**B****CD163**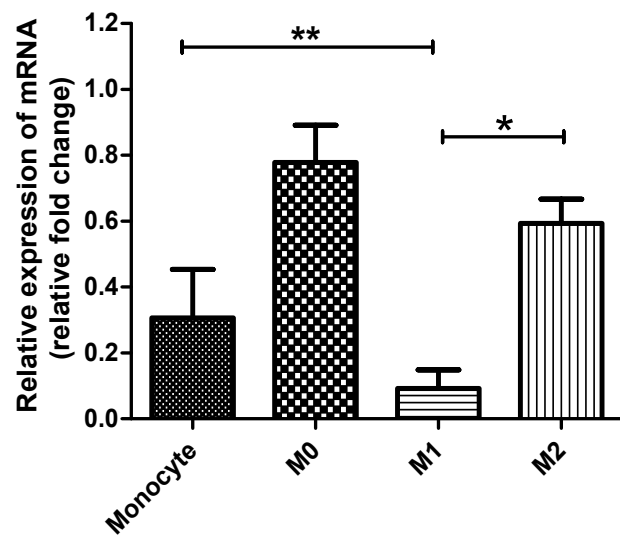**C****CXCL10**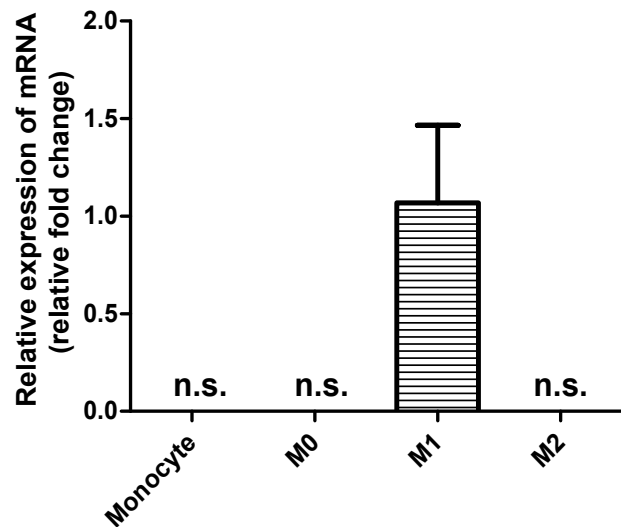**D****CCL18**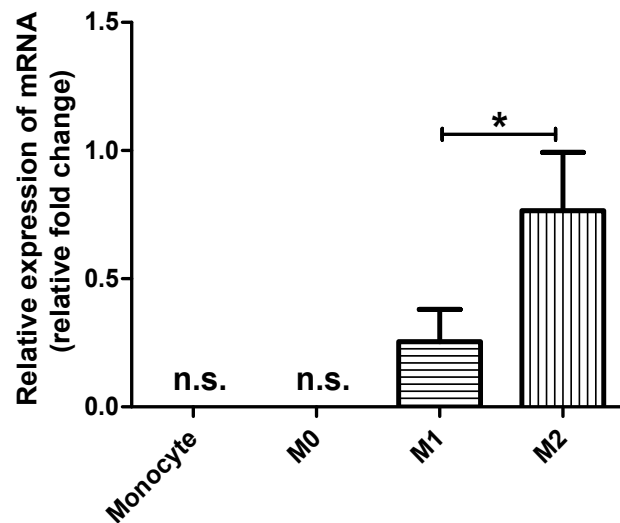

Supplement: S1 Fig — Peripheral blood monocytes, M0 macrophages, and M1 and M2 polarized macrophages were harvested and RNA extracted. mRNA expression of the M2 markers CD206 and CCL18, CD206, considered both an M2 and a macrophage marker, and of the M1 marker CXCL10 were analyzed by RT-qPCR. The expression of each marker was normalized to GAPDH in each group and then calculated as fold change against the expression of monocytes. Data represent the mean and standard deviation of three independent experiments. *P<0,05 **P<0,01 *** P<0.001 (ANOVA with Bonferroni post hoc in A and B and Student’s t-test in D were performed). (PDF) [file pone.0193643.s001.pdf]

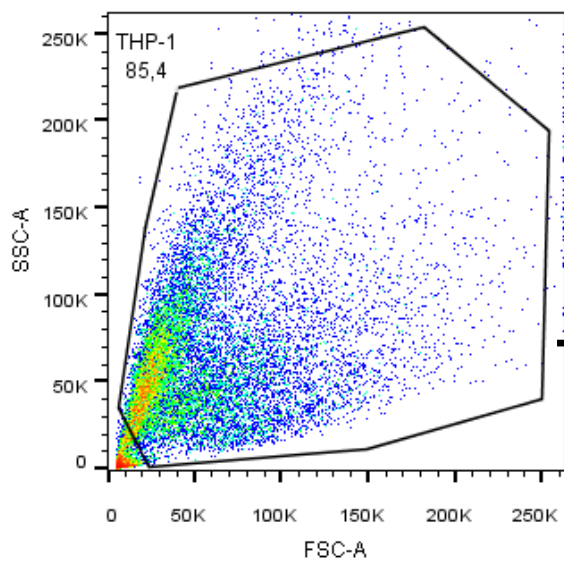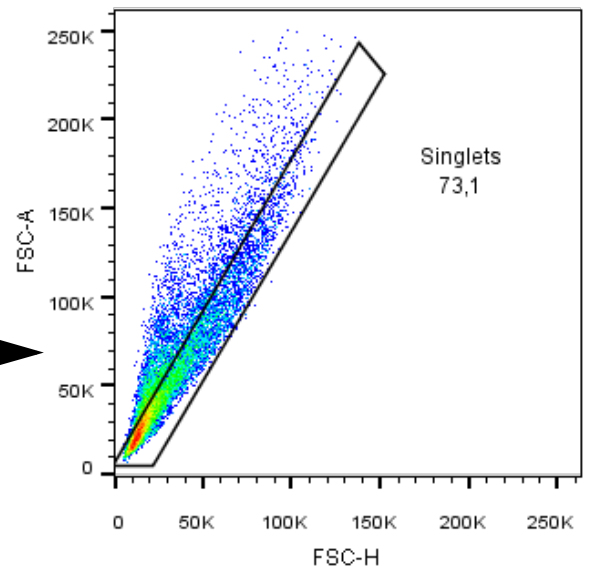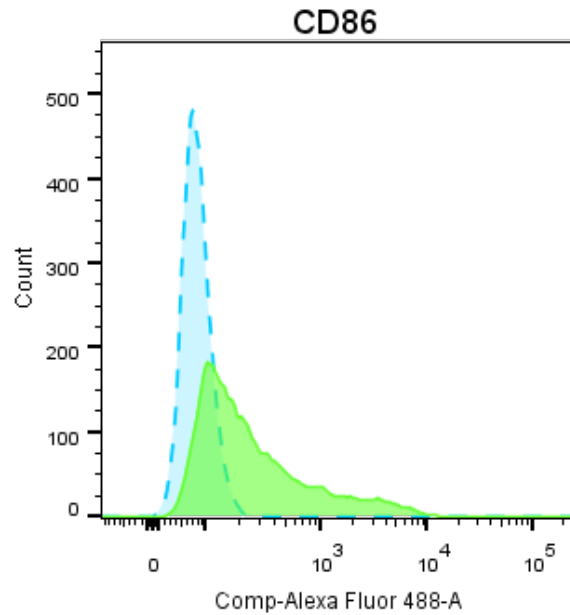

|  | TUBE NAME       | Count | Median : Comp-Alexa Fluor 488-A |
|--|-----------------|-------|---------------------------------|
|  | Macrophage      | 8546  | 240                             |
|  | Isotype Control | 8696  | 95,8                            |

Supplement: S2 Fig — Cells were first gated for monocytes or macrophages (SSC-A vs FSC-A) and then for singlets (FSC-A vs FSC-H). The expression of different markers (CD11b, CD14, CD163, CD86 or CD206) were then analyzed in the singlets gate by measuring their median fluorescence intensity. The gating strategy for CD86, representative of all the markers analyzed, is shown. (PDF) [file pone.0193643.s002.pdf]

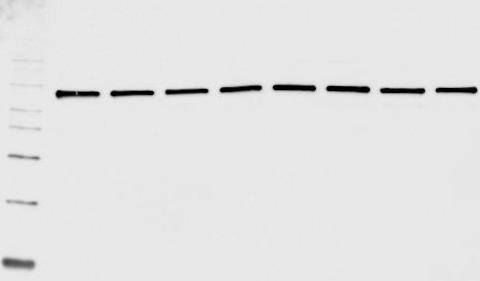

Supplement: S3 Fig — Uncropped Western blot images corresponding to figures in the main manuscript. (ZIP) [file pone.0193643.s003.zip › WB runs/Fig2 HSC70.jpg]

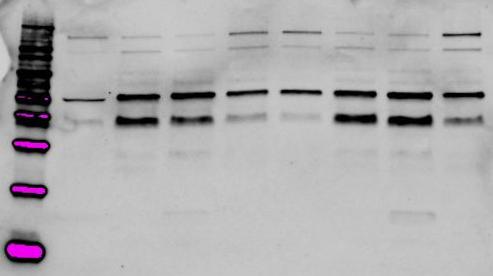

Supplement: S3 Fig — Uncropped Western blot images corresponding to figures in the main manuscript. (ZIP) [file pone.0193643.s003.zip › WB runs/Fig2 NAIP-J2.jpg]

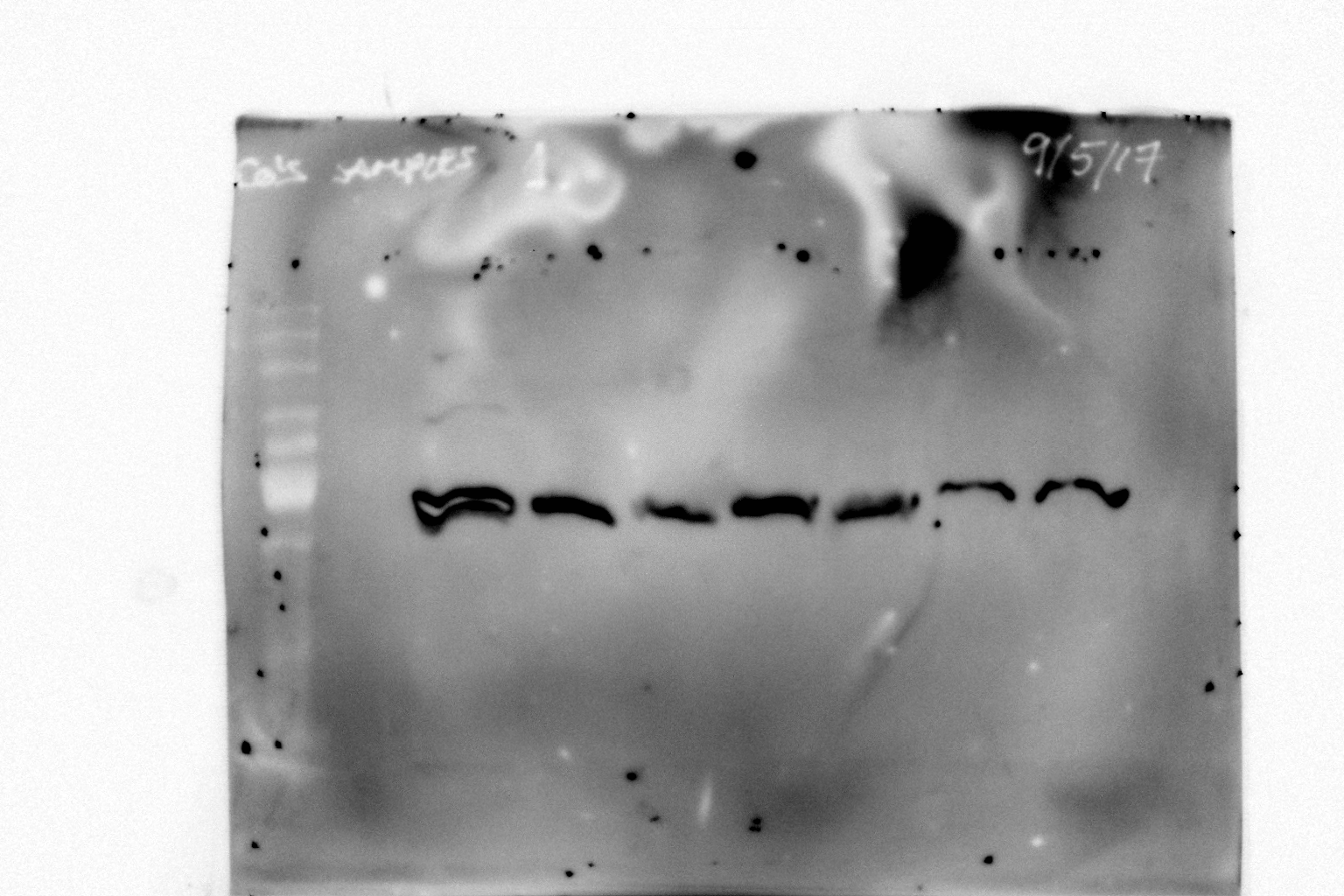

Supplement: S3 Fig — Uncropped Western blot images corresponding to figures in the main manuscript. (ZIP) [file pone.0193643.s003.zip › WB runs/Fig3 HSC70.bmp]

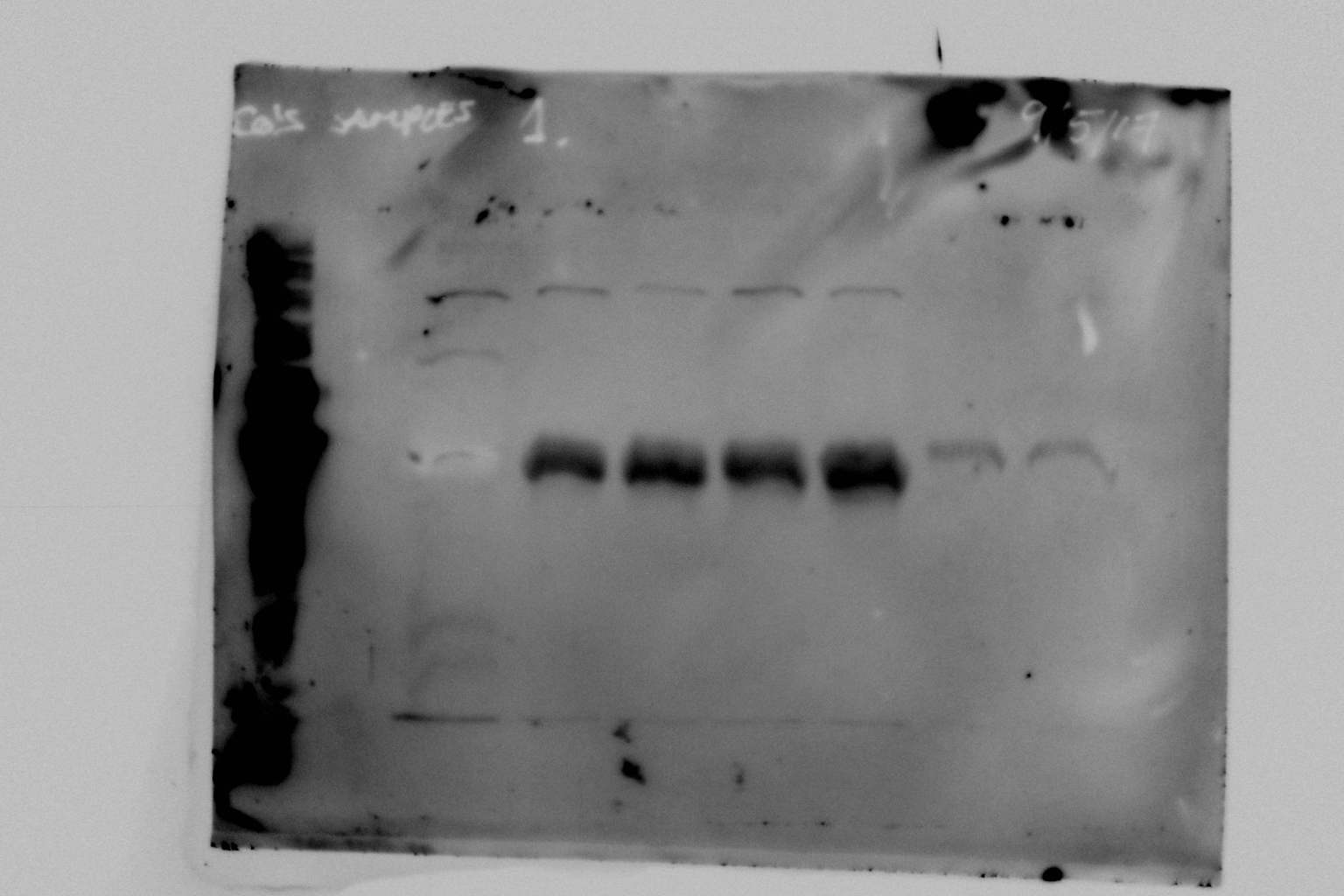

Supplement: S3 Fig — Uncropped Western blot images corresponding to figures in the main manuscript. (ZIP) [file pone.0193643.s003.zip › WB runs/Fig3 NAIP-J2.bmp]

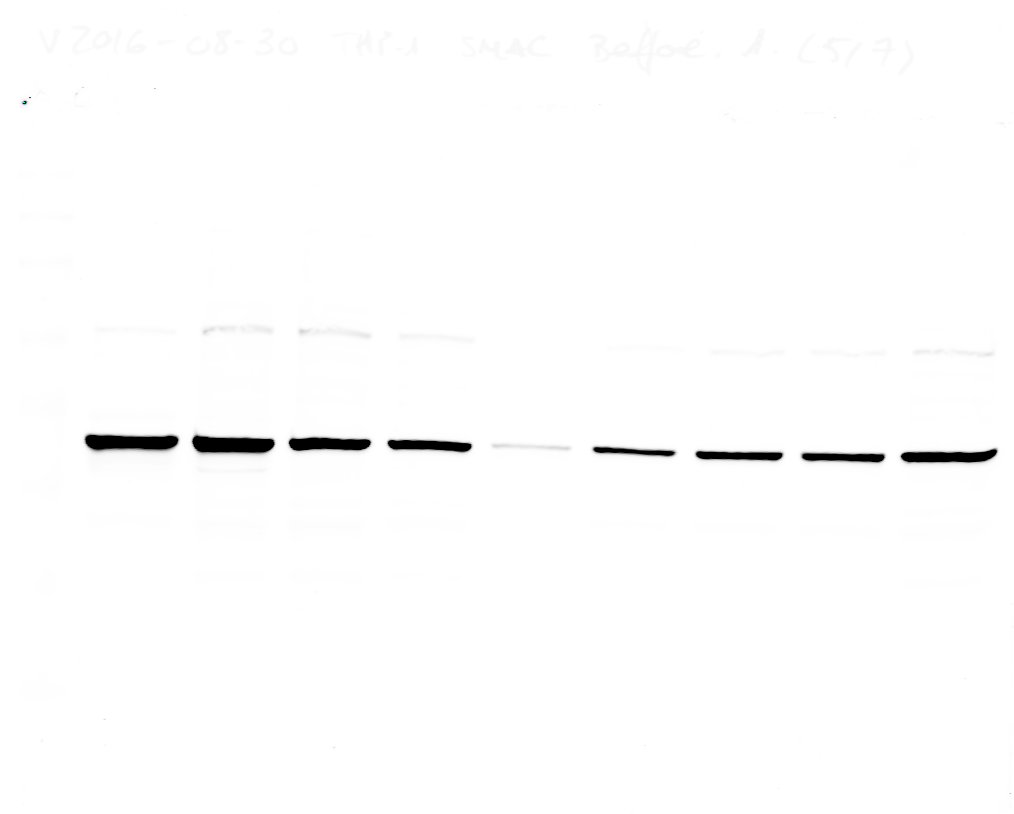

Supplement: S3 Fig — Uncropped Western blot images corresponding to figures in the main manuscript. (ZIP) [file pone.0193643.s003.zip › WB runs/Fig4 HSC70.png]

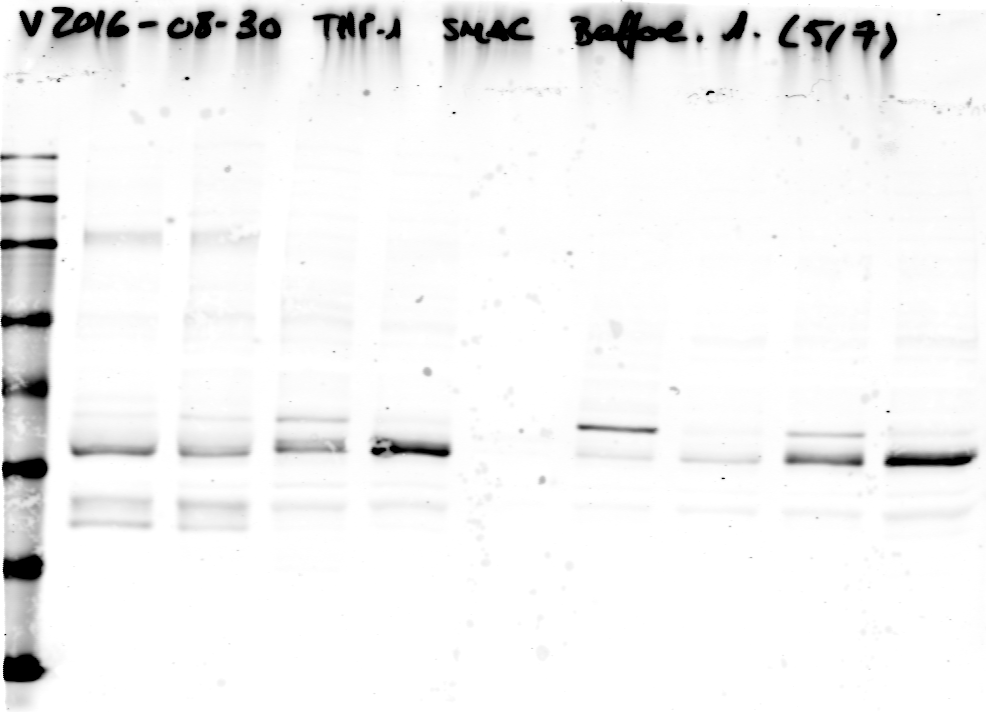

Supplement: S3 Fig — Uncropped Western blot images corresponding to figures in the main manuscript. (ZIP) [file pone.0193643.s003.zip › WB runs/Fig4 RIAP1.png]

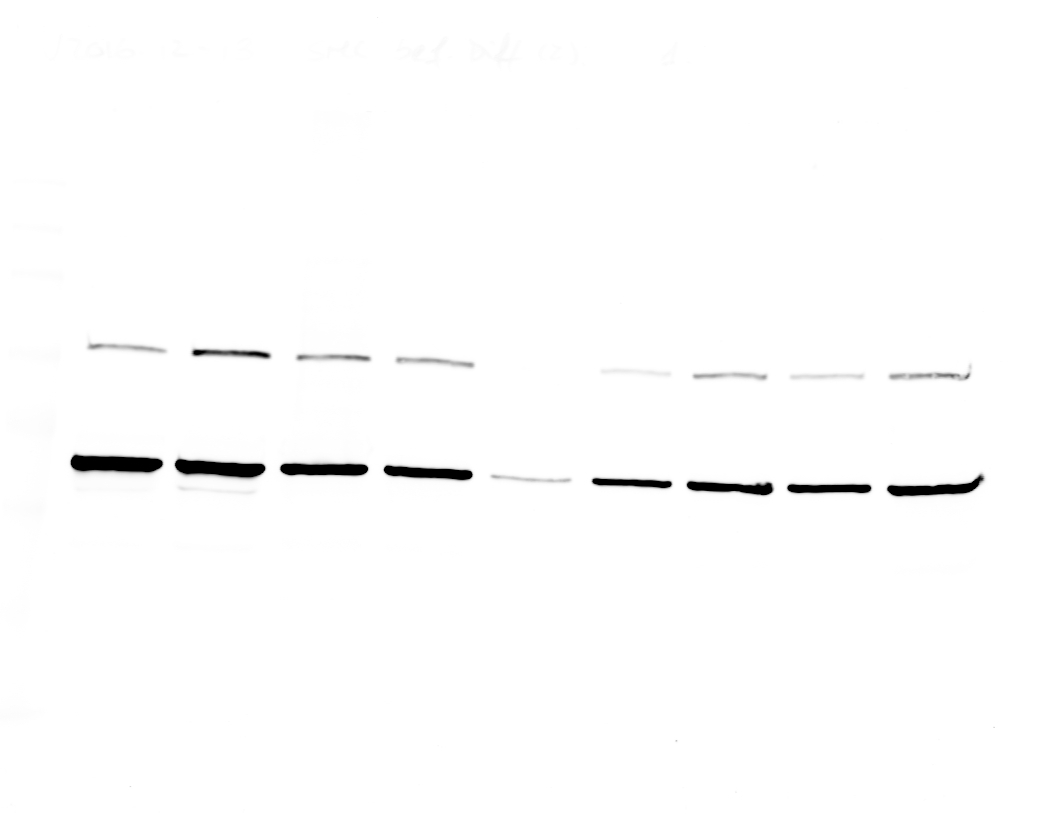

Supplement: S3 Fig — Uncropped Western blot images corresponding to figures in the main manuscript. (ZIP) [file pone.0193643.s003.zip › WB runs/Fig5 naip HSC70.jpg]

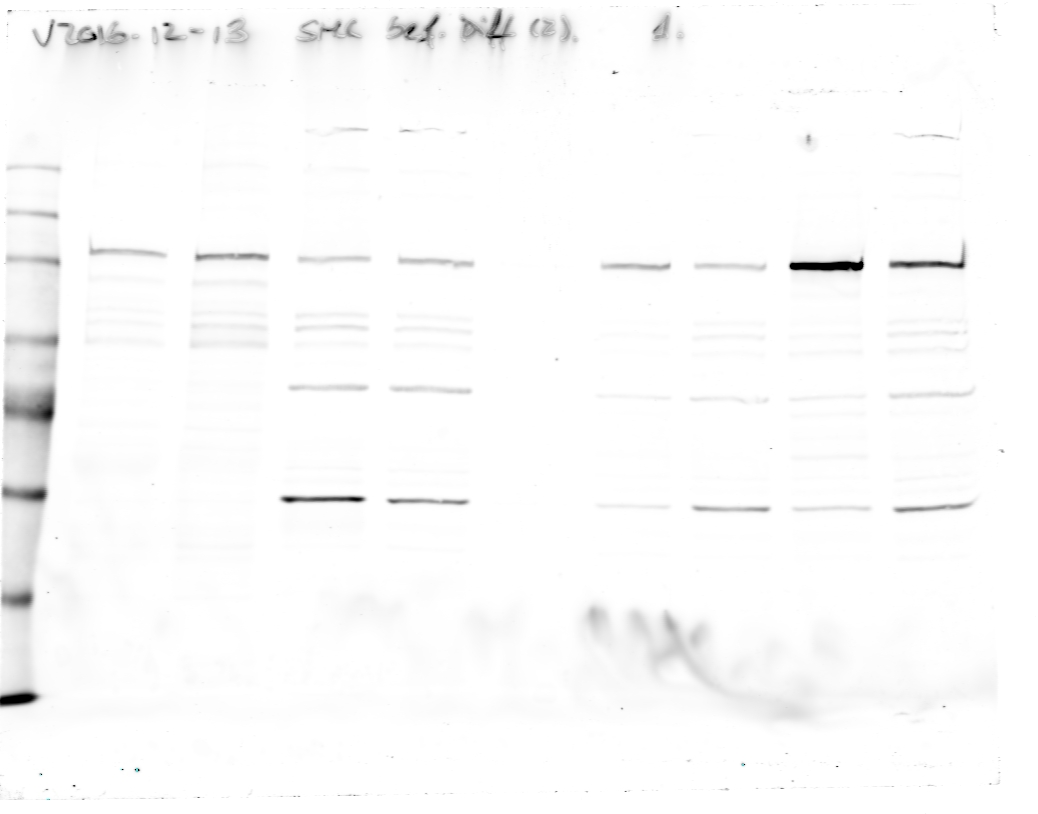

Supplement: S3 Fig — Uncropped Western blot images corresponding to figures in the main manuscript. (ZIP) [file pone.0193643.s003.zip › WB runs/Fig5 NAIP-J2.jpg]

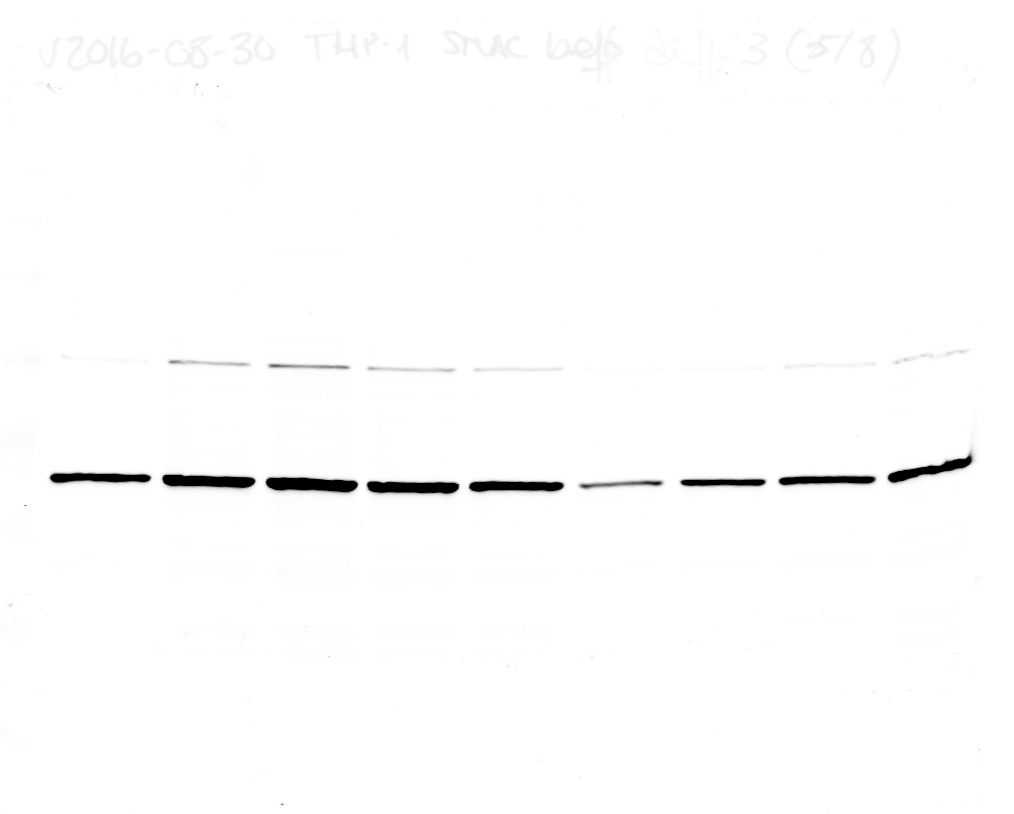

Supplement: S3 Fig — Uncropped Western blot images corresponding to figures in the main manuscript. (ZIP) [file pone.0193643.s003.zip › WB runs/Fig5 riap1 HSC70.png]

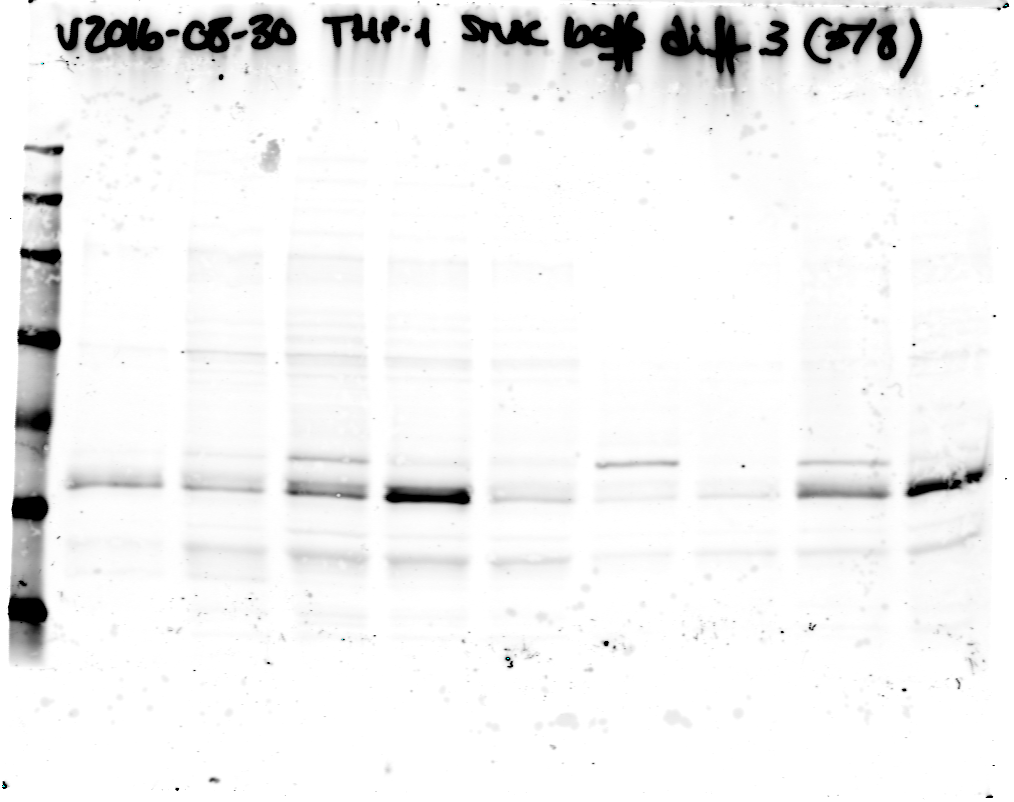

Supplement: S3 Fig — Uncropped Western blot images corresponding to figures in the main manuscript. (ZIP) [file pone.0193643.s003.zip › WB runs/Fig5 RIAP1.png]
